# New Life Through Disaster: A Thematic Analysis of Women's Experiences of Pregnancy and the 2016 Fort McMurray Wildfire

**DOI:** 10.3389/fpubh.2022.725256

**Published:** 2022-05-13

**Authors:** Ashley Pike, Cynthia Mikolas, Kathleen Tompkins, Joanne Olson, David M. Olson, Suzette Brémault-Phillips

**Affiliations:** ^1^Corbett Hall, Faculty of Rehabilitation Medicine, University of Alberta, Edmonton, AB, Canada; ^2^Faculty of Rehabilitation Medicine, Heroes in Mind Advocacy and Research Consortium (HiMARC), University of Alberta, Edmonton, AB, Canada; ^3^Edmonton Clinic Health Academy, Faculty of Nursing, University of Alberta, Edmonton, AB, Canada; ^4^Faculty of Medicine, Heritage Medical Research Centre, University of Alberta, Edmonton, AB, Canada

**Keywords:** stress, resilience, pregnancy, natural disasters, factors of resilience

## Abstract

**Background:**

On May 3, 2016, residents of Fort McMurray Wood Buffalo, Alberta were evacuated due to an uncontrolled wildfire. The short-notice evacuation had destabilizing consequences for residents, including changes in routines, loss of control, and increased uncertainty. These consequences were especially detrimental to women who were pregnant or pre-conception during the evacuation. Pregnant and pre-conception women are particularly susceptible to a vast range of negative consequences during and post natural disasters, including elevated stress and higher incidence of pregnancy complications including gestational diabetes mellitus, pregnancy induced hypertension and C-section. The aim of this study was to understand the experiences, perceived stress and resilience of women who were pregnant during the wildfire. As well as to explore potential interventions to promote the health and enhance resilience of pregnant women and to assist in recovery after exposure to a natural disaster or other traumatic events.

**Methods:**

A qualitative thematic analysis of 16 narratives penned by pregnant women and recounted in Ashley Tobin's compilations 93/88,000 and 159 More/ 88,000: Stories of Evacuation, Re-Entry and the In-Between was conducted.

**Results:**

Analysis revealed five key themes: (1) experience of stress responses due to personal and external factors, (2) social connectedness and support as a facilitator of resilience, (3) performance of resilience-enhancing activities, (4) the roles of pregnancy and motherhood in the experiences of loss and resilience, and (5) the importance of home.

**Conclusion:**

Pregnant women have unique barriers that may negatively impact them during a natural disaster or other form of stressful event. They may benefit from assistance with navigating role transition during pregnancy, training in stress management strategies, and writing interventions to build resiliency and begin the process of recovery from trauma.

## Introduction

An unrelenting, unpredictable wildfire engulfed Fort McMurray Wood Buffalo (FMWB), Alberta in 2016, prompting the short-notice city-wide evacuation of approximately 88,000 people (1). Beginning on May 1, 2016, the fire remained uncontrolled until July 5, 2016, and burned nearly 570,000 hectares of land. An estimated $6 billion in commercial and personal damages resulted due to the destruction of approximately 2,400 homes and businesses (2). Residents in specific areas were able to return to their homes in June 2016, while others who lost their homes were unable to return until the spring of 2017.

The economic impact on the community was not the only destabilizing consequence residents of FMWB experienced. Residents received little warning in advance of the wide-scale evacuation, which resulted in significant disruption of routines, loss of control, and increased uncertainty, potentially increasing stress and anxiety (3). Despite the disruption, affected individuals must find a way to continue moving forward. In 2016, 55,595 babies were born in Alberta. While it is unknown how many of these women and infants were impacted by the FMWB wildfire specifically, pregnant women experiencing disasters are in a unique position, as unexpected traumas can impact their developing fetus (4).

### Impacts of Disaster Exposure to Individuals

The outcomes of disaster exposure are immensely complex and may take months or years to conclude (5). Individuals exposed to disasters may experience increased observable stressors and develop subjective beliefs regarding cause and effects of the event (5). Research into impacts of natural disaster exposure consistently indicates increased disturbances in social and psychological well-being, frequently influenced by an individual's coping styles in response to stressful events (6). Women and children exposed to natural disasters experience increased stressors and a vast range of negative consequences including mental health concerns such as post-traumatic stress disorder (PTSD) (7–9).

Specific to FMWB, research by Moosavi and colleagues (10) found increased rates of probable PTSD, depression and anxiety as compared to self-reported prevalence prior to the wildfire in Fort McMurray Primary Care patients. Brown et al. (11) indicated similar increases in mental health concerns, with nearly half of student study participants meeting criteria for one or more probable diagnoses including depression, anxiety, PTSD or alcohol/substance abuse.

Natural disasters can disrupt an individual's occupational performance and ability to perform daily activities within their environment. Experiencing a natural disaster during pregnancy adds a layer of complexity. An environmental event may upset a pregnant woman's routines, roles, and occupations during a very significant and meaningful time. Additional barriers, such as limits to mobility in pregnancy, may enhance the challenges caused by an environmental event. As stress due to unexpected trauma during pregnancy can have long-term impacts that can compound across generations (4), reducing these negative consequences is of critical importance. When combined with the vulnerability of exposure to a natural disaster, the intersection between pregnancy and trauma creates a form of double jeopardization (12). As such, pregnant women who experience a natural disaster represent a vulnerable population, highlighting the importance of understanding women's experiences of pregnancy during traumatic events. Knowledge about how the women experienced stress and pregnancy throughout wildfire and evacuation can inform interventions to support women who have experienced trauma during pregnancy, as well as pregnant women who may experience a future traumatic event.

### Prenatal Experience

While pregnancy and childbirth are incredibly significant, personal, and spiritual experiences (13), they cause changes in one's body, mind, relationships and routines and are associated with risks to a woman's physical, mental, and emotional well-being. Physical risks and complications include anemia, gestational diabetes mellitus, and hypertension (14). Mental health challenges can also arise. These health risks can impact the developing fetus, with the average rate of fetal deaths per 1,000 total births ranging from 7.5 to 8.1 in 2005–2014 (15). Preterm births during this same period were reported to occur in approximately 8 percent of live births, and very early births (prior to week 32) were found to make up 1.2 per 100 live births.

In addition to changes in physical appearance, women may also experience changes in their identity, roles and occupations (16), especially during their first pregnancy (17). Changes in relationships may also occur as a new understanding of self and the roles of others are developed (18). Pregnancy may facilitate greater intimacy in significant relationships or become a source of tension. Roles, occupations, and environments are important during pregnancy and are significant to understanding women's experiences of a natural disaster and their subsequent loss and grief. Pregnancy represents a transitional period (19), despite the continuation of daily routines and occupations (13). Engaging in normal routines and occupations, however, may become challenging during pregnancy and the post-partum period (19), with mobility and sleep becoming challenging. Experiencing a natural disaster during pregnancy can further alter an individual's occupational performance and their ability to perform daily activities. In addition to the changes in occupation that women experience during pregnancy, they also take on new occupations specific to pregnancy and birth, including preparing for delivery, transitioning to family expansion, and caring for the baby (19, 20).

### Prenatal Maternal Stress and Child Development

Experiencing stress during pregnancy increases the risk of long-term effects to both mother and child (21–24). Research further indicates that parental trauma can be transmitted to offspring, with outcomes dependent on a complex interplay of biological, familial, and cultural systems (25). Pregnancy outcomes of women who experienced Hurricane Katrina showed a correlation between elevated stress and higher incidence of pregnancy complications including gestational diabetes mellitus, pregnancy induced hypertension and C-section (26). Stress responses such as loss of sleep or appetite, which may affect maternal health, can also be attributed to environmental events (27).

Objective prenatal maternal stress (PNMS) can affect the development of the baby *in utero* and have negative impacts on birth weight (24, 27). In an Australian study on birth outcomes following a bush fire, O'Donnell and Behie (22) found an increase in preterm births and babies born with low birth weight in comparison to previous years. PNMS has also been shown to impact childhood development. Research on the impacts of PNMS experienced by pregnant women during a flood was found to be negatively correlated with infant problem solving and personal-social skills at 6 months of age (23). Project Ice Storm (28), a research initiative following the Quebec ice storms of 1998, examined the long-term implications of prenatal exposure to a natural disaster on childhood development. PNMS was found to be related to decreased bilateral coordination and visual motor integration in children at five and a half years of age (21).

The impacts of maternal stress may be influenced by how the stress is perceived. Several studies have explored this relationship through examining the impacts of objective or subjective stress on both parent and child. Research by Cao et al. (21) found high maternal subjective distress was associated with greater negative impacts on motor function in children. Conversely, low subjective distress showed significant harmful effects when stress was high (21). Simcock et al. (23) found that objective maternal stress measures were more predictive of decreased problem-solving skills in infants at 6 months.

Based on research with rats, Yao et al. (4) suggested that experiencing a stressor, including exposure to natural disaster during pregnancy, may be predictive of chronic disease in later life. This study also found that prenatal stress compounded across generations and was a determinant in future maternal health (4). As stress due to unexpected trauma during pregnancy can have long-term impacts that compound across generations (4), reducing these negative consequences is of critical importance. Exposure to a natural disaster *in-utero* may affect infant and childhood development, create long-lasting impacts on well-being, and impact future maternal health of infant girls. Understanding the experience of coping with stress during pregnancy throughout the wildfire and evacuation can inform interventions to support women who have experienced trauma during pregnancy, as well as pregnant women who may experience a future traumatic event. These findings substantiate the importance of further research in this field.

### Potential Intervention

Given the potential long-term physical and mental health outcomes trauma survivors and their offspring may encounter (25), it is imperative that potential interventions be explored. Chen et al. (6) found that elements of emotion-focused coping, including positive reframing, acceptance, and emotional support were protective factors, reducing the subjective distress for pregnant women in flood-related natural disasters. Writing interventions provide an adaptive method for navigating life transitions and coping with traumatic and stressful events and can portray features of emotion-focused coping throughout narratives (29, 30). A meta-analysis on writing therapy found writing to be an evidence-based and efficient intervention for individuals experiencing post-traumatic stress (31). Horsch et al. (32) found that an expressive writing intervention was linked with decreased symptoms of depression and PTSD in mothers of preterm infants. Writing is efficient as it is cost-effective and does not require as much therapy time as other interventions (31, 32). Studies have demonstrated that the task of writing is an effective intervention during times of stress. In a meta-analysis on writing therapies, van Emmerik et al. (31) indicated writing was an efficient and evidence-based intervention for populations experiencing post-traumatic stress, with decreased symptoms of post-traumatic stress and depression resulting. At times expressive writing is an intervention prescribed for persons experiencing times of stress and at other times, they spontaneously take up writing as a way of recording their thoughts and feelings in the moment.

## Objective

This study sought to understand the experiences of women who were pregnant during or shortly following the Fort McMurray wildfire, particularly the perceived stress and resilience they experienced during the wildfire, evacuation and re-entry. This knowledge may help inform future interventions to enhance resilience, promote health, support post-traumatic growth and reduce the harmful effects of natural disasters and other traumatic stressors.

## Methods

A qualitative secondary analysis of expressive writing using thematic analysis was conducted examining narratives written by 16 women who were pregnant during the 2016 FMWB wildfire. The narratives were captured in two books compiled by Ashley Tobin – 93/88,000 (33) and 159 More/ 88,000: Stories of Evacuation, Re-Entry and the In-Between (34). Convenience sampling was utilized by the author to obtain the written entries. Thematic analysis (deductive and inductive) was conducted using methods adapted by Braun and Clarke (35). Graduate-level researchers transcribed the excerpts into NVivo 12. Data was coded and themes identified. All data was coded twice to increase consistency in the process and discrepancies were resolved through discussion. Emerging themes and patterns were analyzed, consolidated and refined and supporting quotes isolated.

## Results

Based on the analysis of the women's narrated experiences of pregnancy during the evacuation, five key themes emerged: (1) experience of stress responses due to personal and external factors, (2) social connectedness and support as a facilitator of resilience, (3) performance of resilience-enhancing activities, (4) the roles of pregnancy and motherhood in the experiences of loss and resilience, and (5) the importance of home. Further descriptions of themes with supporting quotes are found in **Tables 1**–**5**.

### Theme 1: Experience of Stress Responses Due to Personal and External Factors

Throughout the fire and evacuation, women experienced varied stress responses during and/or following the event, including significant emotional reactions and lack of sleep.

“The fire is all around us. It's like we were watching a movie or were stuck in a nightmare. It's like our whole world is falling down on us. We can feel the heat from the fire at our house and ash is falling from the sky. We're all in shock and can't move.” [P165].

Several women also experienced pregnancy complications potentially related to heightened stress such as intrauterine growth restriction, C-section, and preterm births.

“My OBGYN finally explained to me that extreme stress can cause a baby to stop growing. There are medical terms that I researched and googled like crazy. Intrauterine growth restriction, placental insufficiency, small for gestational age.” [P228].

The women also shared stressors and fears related to the event included separation from family members or significant others; fear of losing their homes; fears of losing possessions; fears of running out of gas; waiting in traffic; and well-being of their baby. These stressors and fears often highlighted the women's values, such as connection with family, safety of their children, and protection of their homes, representing uncontrollable circumstances external to the individual.

“We headed down the one way on the wrong side of the road towards the camps. The highway was gridlocked. I can remember my husband saying, “One quick shift of the wind + were all wiped out.” Pure terror set in as we waited in traffic for 2 hours.” [P97].

Frequently, interactions between pregnancy and the environment served as an additional barrier to evacuation as women experienced difficulties including limited physical capabilities, the need for washroom facilities, or fatigue. While not atypical to pregnancy, these limitations created additional challenges for women during evacuation. Proximity to labour added additional uncertainty and stress (see Table 1).

**Table 1:** Theme 1: Experience of stress responses due to personal and external factors.

**Themes**	**Findings and supporting quotations**
1.1 Stress Responses were unique to the individual, but a shared experience.	Experiencing significant emotional responses. “*The fire is all around us. It's like we were watching a movie or were stuck in a nightmare. It's like our whole world is falling down on us. We can feel the heat from the fire at our house and ash is falling from the sky. We're all in shock and can't move.”* “*They told me to head to [company], and I would be able to at least get an EMT... to assist me. I just started to cry… in total disbelief of what was happening. It seemed unreal.”* “*Being under mandatory evacuation meant to me that I had to pack my stuff and get the hell out of there. I closed the windows, turned off the fans and AC, and grabbed the weirdest mix of stuff ever in my bag. I dumped the contents of our safe into my suitcase, the oddest mix of clothes with no underwear or socks, my secret cash stash, some water, a knitting project, a flask of water, and my husband's birthday presents.”* “*As we started to get closer to town, it hit me. This is real life. My sister-in-law called back and said, “Don't panic, stay calm. We can't have you go into labor.” I was doing fine until that call. I completely lost it.”* Loss of sleep and focus. “*We were all so tired but couldn't sleep. Thinking about all the things we left behind and would never see again.”* “*I thought I was OK. I was worried about my husband, I had dreams where he was being burned alive along with my unborn baby. He never stopped working and I didn't get to speak to him properly until May 5th. I was just glued to my phone and the TV, like a zombie.”* Pregnancy/labor complications. “*My OBGYN finally explained to me that extreme stress can cause a baby to stop growing. There are medical terms that I researched and googled like crazy. Intrauterine growth restriction, placental insufficiency, small for gestational age.”*
1.2 Common fears were experienced.	Separation from family/significant others. “*That moment I had to leave my daughter + husband behind so I could get to Albian Sands quicker, it made me feel sick.”* “*I freaked out. I was pregnant and alone with my daughter, literally running for my life down Abasand hill. My husband was at work trying desperately to come to us. I felt there was a possibility I may never see him again until eternity.”* Losing their home and possessions. “*While waiting in line, I break down bawling my eyes out and my fiancé says “Are you okay? What's wrong?”. Through my tears I say, “That could be the last time we see our home. We have a 19-month-old little girl, I'm 31 weeks pregnant and right now we may be homeless.”* “*As we are coming up Beacon Hill I couldn't even look out the window. I'm sitting there thinking “what if we don't get to go back? I forgot so much stuff!” This is our home we could be losing along with thousands of other people.”* “*That's when everything sank in. Will I see my home again? I didn't grab any baby items! I just had my baby shower!!”* Transportation. “*We all make the decision to try and head south once we get the chance. We knew north would not have enough accommodations for everyone and that we definitely wouldn't have enough gas to get us there.”* “*As I was figuring everything out, I realized I had only 88 km to the tank. What if I get stuck in traffic? How would I get out of here?”* “*We headed down the one way on the wrong side of the road toward the camps. The highway was gridlocked. I can remember my husband saying, “One quick shift of the wind + were all wiped out.” Pure terror set in as we waited in traffic for 2 hours.”* The well-being of the baby. “*We weren't sure what our plan was yet but it didn't take long to decide the new smoky conditions wouldn't be great for our 48-hour old baby.”* “*The first night, I was in so much pain from my C-section and was still trying to come to terms with what we just went through. I spent the night knelt on the floor hunched over the bed, crying, trying to sleep, and worried that something was going to happen to my baby.”*
1.3 Pregnancy possessed an additional barrier to evacuation.	Pregnancy was an added complication during evacuation. “*She'd asked if I could get on a flight and at first was told ‘Sorry, she's too pregnant to fly…' CUE meltdown number two. How on Earth was I going to have a baby in a CAMP?!”* “*[Driving 6.5km] took us 45 mins, that's when I noticed something felt different the pains in my back + stomach were getting closer I started to watch the clock...I called my husband and told him we needed to stop and talk to the RCMP, at this time noticing everything around us was on fire Husband: no we can't stop here. Me: Yes my contractions are 4 mins apart! Husband: No your joking right?! Are you sure?”*

“She'd asked if I could get on a flight and at first was told ‘Sorry, she's too pregnant to fly...' CUE meltdown number two. How on Earth was I going to have a baby in a CAMP?!” [P64].

### Theme 2: Social Support Was a Facilitator of Resilience

Connection with loved ones was an important facilitator of resilience as women received support from family and significant others during and after the evacuation process, with one participant writing,

“My husband helped me through each day as I battled my emotions. He was simply amazing <3 But it's still hard.” [P81].

Writing expressed the value of relationships and togetherness with family members and spouses or partners. Whereas separation from significant others was a source of fear and stress, reunions with loved ones eased stressful emotions (see Table 2). Connection and support from the community was also an important facilitator of resilience during women's experiences of the fire and evacuation. Pregnant women received assistance from service providers and health care workers who examined and offered escorts to help women reach safety quickly. The women also were offered compassion and kindness from others, with one woman and her family being allowed into a camp after it was full. One new mother with a one-day old baby shared:

**Table 2:** Theme 2: Social support was a facilitator of resilience.

**Themes**	**Findings and supporting quotations**
2.1 Support from significant others facilitated resilient attitudes.	Significant others as a source of support. “*I closed the door and instantly started crying. I was 8 months pregnant. I had no idea what to pack so I took a step back and thought to myself...I need to call my husband so he can come home and help me.”* “*My husband helped me through each day as I battled my emotions. He was simply amazing <3 But it's still hard.”* Connection with loved ones as a stress reducer. “*As soon as I seen my sister we both started to cry, I was happy to be somewhere safe and with family.”* “*[My husband] abandoned his vehicle and walked the rest of the way to us through the thick smoke. When I finally saw him walking through the smoky parking lot at the hospital, it was better than my wedding day. I could breathe again. I felt safe. With flames all around us I wasn't scared. I knew he would keep us safe.”*
2.2 Support from others/ community facilitated resilient outcomes.	Acts of kindness as sources of support. “*The workers...were all staring at me and felt so sorry this was happening while I was in labor. They were very nice and offered us their rooms, and even offered to take our pets and look after them for the night.”* “*Two guys asked us if we needed help with our bags. We told them we didn't have a ticket + were on standby. They immediately handed both of us their tickets, then helped us carry our bags to the plane. Whoever these two strangers are...thank you, from the bottom of my heart.”* Provision of needs as sources of support. “*We had so many offers for places to stay, food, and even the doctor that checked the baby offered us to stay with her family!”* “*People were helping us in any way they could! A kind man in Spruce Grove took my mom and sister out shopping, and a sweet old lady handed us money after hearing our story. I was in awe of all the compassion.”*

“Two guys asked us if we needed help with our bags. We told them we didn't have a ticket + were on standby. They immediately handed both of us their tickets, then helped us carry our bags to the plane. Whoever these two strangers are...thank you, from the bottom of my heart.” [P116].

Strangers made significant personal sacrifices from allowing participants to move in front of them in traffic or giving up flight tickets to ensure safety was reached. Women experienced generosity from others through donations and offers of places to stay. Women expressed feeling cared for and overwhelmed by the community support.

### Theme 3: Performance of Resilience-Enhancing Activities

Women described engaging in resilience-supporting behaviours. Across the narratives, they commonly wrote of practicing gratitude and framing circumstances positively. Women expressed gratitude for those who assisted them during the evacuation as well as during labour and delivery. They were thankful to the first responders who fought the fire, and to the individuals who provided support after the event.

“[T]he kindness and generosity of strangers and the true efforts of our first responders will never be forgotten. There are no words to express our gratitude and love for you all.” [P165].

Women expressed gratitude for significant others and extended families who kept them grounded during the evacuation and the aftermath. Furthermore, they identified positive aspects of the experience, for example, recognizing

“Our daughter will have a crazy birth story to tell when she gets older!” [187].

Finally, the pregnant women expressed pride and solidarity with their community (see Table 3), reflecting on their pride in how the citizens of FMWB successfully navigated difficult circumstances.

**Table 3:** Theme 3: Performance of resilience enhancing activities.

**Themes**	**Findings and supporting quotations**
3.1 Gratitude and thankfulness were expressed.	Thankfulness for hospital staff, first responders, and other service providers. “*Thank you from the bottom of my heart to the emergency staff at the [hospital], especially the PICU ward, and the respiratory staff for helping our sweet boy in his fight”* “*The kitchen was filled with staff working overtime trying to ensure everyone got a good meal...- thank - you [company name]!”* “*Thank you to the emergency crews for leaving your families to save our city.”* “*We would like to also thank all the emergency personnel from all around Canada for helping our city when we needed it most.”* Gratitude for kindness from strangers. “*I get out of my SUV and ask the guy behind me. Please can my husband jump ahead of you, I'm worried being 3 days overdue we need to be together. He said “of course“. Thank you whoever you are - the man from Tower Rd hauling your camper.”*
	“*Even though this day will always be engraved in our memories, the kindness and generosity of strangers and the true efforts of our first responders will never be forgotten. There are no words to express our gratitude and love for you all.”* Gratitude for significant others. “*And to my husband; thank you for being my rock, my strength, my shoulder to lean on and my person to hug. Thank you for keeping our family safe”*. “*I will be forever grateful to my family and my husband for keeping me calm and for getting us to safety.”* Thankfulness for positive outcomes. “*Although being loaded on a city bus 5 hours after a c-section with newborn twins wasn't ideal - I will always be grateful for the care we were given and part of me will always wonder how much worse it might have been if I had been evacuated at 38 weeks pregnant with breech twins and gone into labor.”*
3.2 Circumstances were framed positively.	Positive feelings toward outcomes. “*We lost all of our material possessions that day but in the months to follow we were blessed far beyond what we could have imagined.”* “*We were one of the lucky ones that didn't lose our home. I thank god every day for: keeping everything we have worked so hard for and everyone safe, for not going into labor due to all the stress, and that my fiancé was on night shift that day because without him I'm not sure how our story would've turned out.”* Positive feelings in difficult situations. “*It wasn't the best living arrangement but it could have been worse too.”* “*Our daughter will have a crazy birth story to tell when she gets older!”* Pride in community resilience. “*It is amazing to see how strong our city has been since the Beast, and the compassion we've all received...It makes me so proud to say that Fort McMurray is my home.”* “*I am proud to say that I love the community that I live in. I will always be Fort McMurray strong.”*

### Theme 4: The Roles of Pregnancy and Motherhood in the Experiences of Loss and Resilience

The women identified disruption of transitional changes into new or unique roles, occupations, and interactions with the environment related to pregnancy as a result of the wildfire. Many of the women also noted that pregnancy was a time of excitement and anticipation, indicating

“I decided that morning [May 3] to start bouncing on an exercise ball cause I was anxious to meet my little man” [P67].

Others additionally noted that ordinary routines were also required, including caring for children, household tasks, and external employment. However, these roles and occupations were disrupted by the wildfire, as women expressed feelings of loss and disappointment regarding what they had hoped to experience as a mother and for their new baby.

“We decided to drive through the night South towards Saskatoon. 3 adults, 2 dogs and a 3-day-old baby all crammed into the SUV. Definitely not how I had imagined my first days postpartum.” [P107].

For new mothers, the fire and evacuation acted as a barrier to performing and savouring new mothering occupations. In addition to disappointment in expectations, the wildfire and evacuation acted as a barrier to engaging in mothering roles and occupations, which exacerbated experiences of stress, lack of control, or grief as women sought to provide for their babies' needs. The disrupted environment resulted in barriers to carrying out mothering roles and occupations during the postpartum period. A small number of women noted experiencing a miscarriage during or following the events of the fire.

“As November 21st approaches, I can't help but dwell on this day as it would have been my due date. I would trade spots in the blink of an eye to have my home burnt down if it meant I was still pregnant.” [81].

While it is unknown whether the fire was a cause of the miscarriage, these narratives express tragic losses amidst their experiences of the wildfire related to the lost life and roles. Despite the loss and grief, many of the narratives also related stories of birth, acceptance, homecoming, and demonstrated resilience. These stories bring a sense of restoration with women regaining valued roles, occupations and environments as amidst grief and loss many of the women gave birth, contrasting the horror in the fire and evacuation. Women exhibited resilience in adapting to high-stress environments while maintaining roles and routines of motherhood.

“May 5th up for 24 + hours I needed to rest, but priority #1 car seats for the kids, new baby clothes, diapers etc. we had nothing for the baby” [P252].

They emphasized the roles of provision and protection, finding creative ways to provide for their children's needs (see Table 4).

**Table 4:** Theme 4: The roles of pregnancy and motherhood in the experiences of loss and resilience.

**Themes**	**Findings and supporting quotations**
4.1 Pregnancy was a time of anticipation.	Pregnancy is an exciting time. “*We were scared as first time parents, but so excited for our new adventure.”* Pregnancy as a time of preparation. “*May 1st - I spent the day getting all the last minute details finished. I was due to have our baby girl on May 5th so I knew there wasn't much time left to get everything done. I was organizing her nursery and I was nesting so much that I spent hours scrubbing the grout on the bathroom floor. I just wanted everything perfect for when we brought our first born home from the hospital.”*
4.2 Women performed mothering occupations and routines.	Importance of regular routines. “*I was two days shy of 36 weeks pregnant while I was making dinner for my husband + 2-year-old on the day of May 3rd when people started to voluntarily evacuate from certain areas of the city.”* “*I started with my morning routine of getting our 4-year-old ready for preschool.”*
4.3 Women experienced loss/ disappointment in relation to roles of being pregnancy/ mother.	Feelings of loss regarding the expectations of new motherhood. “*As a new mom, I didn't get to take my baby home. I didn't get to experience that first night at home with the newborn.”* “*We decided to drive through the night South toward Saskatoon. 3 adults, 2 dogs and a 3-day-old baby all crammed into the SUV. Definitely not how I had imagined my first days postpartum.”* Feelings of grief for perceived failure in fulfilling their role as a mother. “*I feel guilty. I couldn't protect my beautiful little baby girl and I failed her when she needed me most to be strong and fine and to hold it together despite everything going on around us. When people make comments about her size, although she is growing and completely healthy, but still petite for her age, I am reactive because it reminds me of my first failure to protect her and keep her safe. My first experiences as a mother are guilt and helplessness that I could not do a better job in protecting her.”*
	Grief over the loss of life. “*I hear people tell their stories of loss, loss of home, loss of items. Some have property damage. As I sit and listen to all these stories, I am weeping on the inside for the loss of my baby. No amount of insurance money or Red Cross funds will fix my loss.”* A loss of pregnancy/labor roles with the experience of miscarriage. “*I asked for no pain medications for the DNC because I wanted to feel the pain of labor.”* “*As November 21st approaches, I can't help but dwell on this day as it would have been my due date. I would trade spots in the blink of an eye to have my home burnt down if it meant I was still pregnant.”*
4.4 Women experienced loss of pregnancy/ mothering occupations.	Concern over abilities to provide for their babies needs. “*As we learned our stay in Edmonton would be longer than planned, I got so depressed and all I could do was cry. I was stressed about not having anything for the baby.”* “*The nurse looked at us + told us our baby girl will be here today...Besides being happy, we were scared she was early. We didn't have anything for her. Not sure what our house situation was gonna be But Ready or not, here she comes!”* Disruption of new occupations related to being a mother. “*I never imagined I would be changing my twin sons' dirty diapers on a city bus. That is probably one of my most vivid memories of the evacuation. I remember thinking that the first day of my babies' life shouldn't be like this”*
4.5 Giving birth brought fulfillment to pregnancy role.	Birth as a sense of meaning contrasting uncertainty and loss. “*After 9 hours of labor & 1.5 hours of pushing our little [baby] arrived @ 3:21 PM. He was so perfect. Everything was perfect.”* “*My family finally arrived around 12:30 PM on May 4th – which was perfect timing. My beautiful son was born at 1:32 PM.”*
4.6 Women adapted mothering roles and occupations.	Roles and occupations were fulfilled. “*We drenched towels and covered our son's car seat in an attempt to repel any possible smoke that may come into the vehicle.”* “*May 5th up for 24 + hours I needed to rest, but priority #1 car seats for the kids, new baby clothes, diapers etc. we had nothing for the baby”* “*I wake up our daughter and try to calmly continue our same routine because I don't want her to be scared. I'm already scared enough for the both of us...The faint smell of smoke is in the air, so I rush to turn our central air off. I didn't want our daughter to smell it.”* Resourcefulness in adapting to new circumstances. “*We didn't have a crib or bassinet for the baby to sleep in, so we did the only thing we could think of - made a bed for her out of a dresser drawer...Its funny how you spend so much time preparing for a baby, getting everything ready, and at the end of the day all we needed was a safe place to sleep, food to eat, we had each other, and we were safe.”*

### Theme 5: The Importance of “Home”

The significance of home was an important theme that demonstrated how women valued and interacted with their environment, as they experienced feelings of fear and loss during the evacuation and experienced resolution in coming home and connecting the concept of home with memories. Mothers frequently narrated the loss of being able to bring their baby home from the hospital, after preparing to do so commenting

“I was so scared, confused and emotional. Our baby was less than a day old. We should be home.” [P116].

Many women experienced fear that they may not see their homes again, bringing a sense of displacement. During homecoming, women experienced strong, predominantly positive emotions, sometimes mingled with fear and anger.

“July 1st finally came and it was the best day ever! I was going home to be with my husband. The drive was quiet. The drive coming into town was full of tears. I was so happy to be home, but so sad for those who lost their homes.” [P66].

For many of the women, coming home seemed to be indicative of circumstances settling. Birth and homecoming often provided a sense of resolution to these events, restoring anticipated roles and routines (see Table 5).

**Table 5:** Theme 5: Place of home was important in women's experiences.

**Themes**	**Findings and supporting quotations**
5.1 The concept of home was valued.	Women valued the concept of home. “*I will never be able to put into words how grateful I am for all first responders. The fire came close, but thank you for saving our home, + our memories.”* “*At 1030 AM on May 5, our baby BOY was born!!! And we can also say we were fortunate enough to bring him home 54 days later.”*
5.2 Women experienced fear and loss surrounding place of home.	Loss of expected experiences. “*As a new mom, I didn't get to take my baby home. I didn't get to experience that first night at home with the newborn.”* “*I was so scared, confused and emotional. Our baby was less than a day old. We should be home.”* Fear of losing homes. “*I could not fathom the idea of leaving our home at that moment of my broken life leaving...if I left...it was as if everything that I knew to be true was now false. But we finally left our home in the early evening on May 3rd”* “*We then lock the door and reluctantly left - the house we'd finally finished”*.
5.3 Significance of homecoming.	Homecoming restored the valued environment of home. “*July 1st, we came back to our home in Fort McMurray with our now 2-month-old son. I remember walking inside and going straight to his room. Everything was just how I left it. I just sat there and held him and cried. This is your room. This is our home. Welcome home, again, sweet boy.”* Women felt strong emotions upon coming home. “*As we made our journey home on June 2nd. I was flooded with raw emotions again...sadness + anger.”* “*July 1st finally came, and it was the best day ever! I was going home to be with my husband. The drive was quiet. The drive coming into town was full of tears. I was so happy to be home, but so sad for those who lost their homes.”*

## Discussion

This study aimed to explore the experiences of women who were pregnant during or shortly following the traumatic 2016 wildfire, with a specific focus on perceived stress and resilience shown through expressive writing. Exploration of the experiences of pregnant women during and following an unexpected traumatic event may provide increased knowledge of how pregnant women experience and respond to stress. Seeking to understand factors of stress and resiliency, along with the roles and occupations important in pregnancy can inform recovery interventions and support resilience-building in prenatal populations in the future.

### Factors of Stress and Resilience

Despite the added complication of pregnancy, participants displayed resilient mindsets and referenced emotion-focused coping through problem solving, gratitude, asking for and accepting help, and maintaining expected roles. Participants indicated they received immense support by others in the community. It was noted in the narratives that they believed this occurred due to their pregnancy. This finding warrants further exploration into the protective factors of community resilience, particularly in terms of prevention of long-term impacts resulting from PNMS. Connection with friends and family, emergency personnel, and supportive community members provided a buffer from elements of stress.

### Roles and Occupations

The person, occupation, and environment all impact a mother's ability to participate in roles and occupations important in motherhood, with stress and self-efficacy being critical to the transition (19). Roles and occupations in pregnancy, birth, and motherhood were important themes that emerged in women's descriptions of the Fort McMurray wildfire, with women noting that the fire acted as a barrier to fulfilling mothering roles and occupations. During a natural disaster, elements within the person, occupation, and environment are subject to disruption and unpredictability, challenging a mother's role participation and occupational performance. Experiencing stress and trauma during pregnancy impact a mother's roles and occupations. As such, understanding the change or loss in roles and occupations are critical for supporting women following unexpected trauma during pregnancy.

### Self-Selected Intervention

The act of writing has been shown to help individuals process traumatic events. In this study, we examined the writings that women shared about their experiences of the evacuation from Fort McMurray and re-entry into the community. While it was impossible to connect with the women who chose to contribute to the book, it is possible to infer from their written text that they used this opportunity to share their thoughts and feelings about their experiences regarding their pregnancies and they were impacted by the wildfire. Through writing, they were also able to explore both the difficult emotions and situations caused by fire and evacuation as well as hopeful and positive outcomes, providing an outlet to process their experiences. Writing may have also provided the women with an outlet to explore important roles, environments, and occupations in pregnancy and birth and to process grief.

### Recommendations

Building resilience in pregnant women prior to experiencing trauma is one approach to reducing the harmful impacts of prenatal maternal stress. Prenatal education may provide an opportunity to teach expectant parents resilience strategies that can be drawn upon during a traumatic event. The FMWB wildfire created a stressful and unpredictable context in which pregnant women experienced significant stress responses. Community and familial support in transitioning roles and routines as disasters unfold is vital to ensuring pregnant women experience as minimal disruption as possible, and this support was frequently documented in the women's narratives.

Coping mechanisms including planning or asking for help were discussed in several narratives, however, physiological stress management behaviors, including breathing or grounding exercises, were rarely discussed. While lack of inclusion does not confirm that these behaviors were not accessed, increased focus on stress management methods may be beneficial during prenatal education. This recommendation supports women through changes during the perinatal period in addition to unexpected stressful or traumatic events. Tragea et al. (36) found significant decreases in perceived levels of stress following implementation of a six-week stress management program for prenatal women, indicating that such strategies may act as a protective factor against harmful effects of stress during pregnancy. An increased ability to cope with stress may help in building resilience in pregnant women. Because natural disasters and other forms of traumatic stress are often unexpected, education on stress management may be beneficial as a widespread intervention for women during the prenatal period. Given the negative effects of stress on birth and childhood outcomes, it is important to support adaptive and healthy ways of responding to stress to build resilience in mothers. Education and health promotion on adaptive coping styles may be beneficial for prenatal women who may experience stress during pregnancy.

### Areas for Future Research

While the risks for women and children facing prenatal stress and trauma are well documented, more research is needed to identify effective interventions to support populations who have experienced an environmental disaster. Current research indicates PNMS impacts childhood development, demonstrating the need for research into proactive resilience-building is critical to assist pregnant mothers with managing stressors and offset potential risks to their infants. Additionally, as women who have experienced a traumatic event may face challenges due to effects of prenatal stress, such as difficulty in adapting to the role of motherhood, research in this area is of critical importance.

### Limitations

There were several limitations to this research study. The data used was not collected by the research team, leaving many aspects of data collection unknown, including when women wrote the narratives following the wildfire. The data contains a small sample size, representing a small number of evacuees. Participants were self-selected, as individuals volunteered to contribute to the writing project. Furthermore, due to the anonymity, researchers were unable to seek participant feedback after analysis was completed. As a result, the findings cannot be generalized to all women who were pregnant during the Fort McMurray Wood Buffalo wildfire.

## Conclusion

Experiencing a natural disaster significantly influences an individual's occupational participation and their ability to recover from the associated trauma. Pregnant women have unique barriers that may negatively impact them during a natural disaster or other forms of stressful event. Support to pregnant women could be offered through assistance with role transition and enabling mothering occupations and routines. Education on stress management and adaptive coping along with facilitating exploration and processing of traumatic experiences through writing interventions can also be used to build resiliency and aid in recovery from traumatic events.

## Data Availability Statement

The datasets presented in this article are not readily available because writing excerpts used in the data analysis were taken from the books 98/88,000 and 159 more/88,000: Stories of Evacuation, Re-entry and the In-between. Requests to access the datasets should be directed to 98/88,000 and 159 more/88,000: Stories of Evacuation, Re-entry and the In-between.

## Author Contributions

AP, SB-P, JO, and DO contributed to conception and design of the study. KT coded the writing excerpts and wrote the first draft of the manuscript. KT, AP, and SB-P performed the thematic analysis. AP, CM, JO, DO, and SB-P wrote sections of the manuscript. All authors contributed to manuscript revision, read, and approved the submitted version.

## Conflict of Interest

The authors declare that the research was conducted in the absence of any commercial or financial relationships that could be construed as a potential conflict of interest.

## Publisher's Note

All claims expressed in this article are solely those of the authors and do not necessarily represent those of their affiliated organizations, or those of the publisher, the editors and the reviewers. Any product that may be evaluated in this article, or claim that may be made by its manufacturer, is not guaranteed or endorsed by the publisher.
